# Membrane Bioreactor Technology for the Development of Functional Materials from Sea-Food Processing Wastes and Their Potential Health Benefits

**DOI:** 10.3390/membranes1040327

**Published:** 2011-10-25

**Authors:** Se-Kwon Kim, Mahinda Senevirathne

**Affiliations:** 1 Department of Chemistry, Pukyong National University, 599–1, Daeyon 3-dong, Nam-Gu, Busan 608–737, Korea; 2 Marine Bioprocess Research Center, Pukyong National University, 599–1, Daeyon 3-dong, Nam-Gu, Busan 608–737, Korea; E-Mail: msaraths@yahoo.com

**Keywords:** membrane bioreactor technology, seafood wastes, functional materials, peptides, chitooligosaccharides, biological activities

## Abstract

Sea-food processing wastes and underutilized species of fish are a potential source of functional and bioactive compounds. A large number of bioactive substances can be produced through enzyme-mediated hydrolysis. Suitable enzymes and the appropriate bioreactor system are needed to incubate the waste materials. Membrane separation is a useful technique to extract, concentrate, separate or fractionate the compounds. The use of membrane bioreactors to integrate a reaction vessel with a membrane separation unit is emerging as a beneficial method for producing bioactive materials such as peptides, chitooligosaccharides and polyunsaturated fatty acids from diverse seafood-related wastes. These bioactive compounds from membrane bioreactor technology show diverse biological activities such as antihypertensive, antimicrobial, antitumor, anticoagulant, antioxidant and radical scavenging properties. This review discusses the application of membrane bioreactor technology for the production of value-added functional materials from sea-food processing wastes and their biological activities in relation to health benefits.

## 1. Introduction

About 78% of the total fish catch in both developing and developed countries is used as human food while the remainder is discharged as waste [1]. Fish processing waste such as skin, bones and fins is about 7.3 million tons/year [2]. There is considerable research that much of this waste, such as gelatin, collagen, fish oil and calcium, could have value as functional or bioactive materials [3,4,5]. However, some of the traditional methods for extracting these materials use organic solvents and acids that may lower the functionality and bioactivity of the native materials. These chemicals could also be harmful since they may accumulate in the environment and cause problems to humans, animals and the environment. On the other hand, enzyme hydrolysis of fish waste has also produced many bioactive compounds. If combined with innovative processing methods such as membrane technology, there could be a new type of bioactive materials and we could avoid some of these limitations. The combination and integration of a bioreactor with membranes is known as a “membrane bioreactor” and is widely used in waste water treatment. Membrane bioreactors could also effectively produce compounds with desirable molecular weights (MW) such as chitooligosaccharides (COS) from chitin wastes, peptides from proteins and polyunsaturated fatty acids (PUFAs) from fish oil [4,6]. Depending on the design of the membrane bioreactor, membranes can be selected and used to separate specific peptides from fish skin, scale gelatin and muscle [7,8].

Many of the compounds produced have shown biological activities including anticoagulation, antioxidant, calcium absorption, antihypertensive, antimicrobial activities [9,10,11,12,13]. This review focuses on the use of membrane bioreactor technology for the production of bioactive compounds from sea-food processing wastes and their biological activities in relation to their health benefits. The bioactive compounds include chitooligosaccharides (COS), bioactive peptides, bioactive lipids and enzymes. The use of membrane bioreactors for waste water treatment from fish-processing plants to reduce the large amount of organic matter [14] and for clarification of environmental effluents [15] is beyond the scope of this review and is not discussed.

## 2. Membrane Bioreactor System

**Figure 1:**
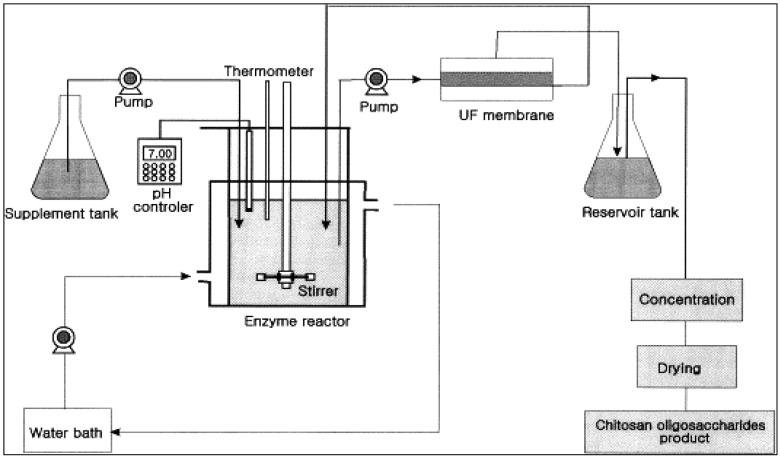
Schematic diagram for the production of chitooligosaccharides in the UF membrane reactor system (Adapted from Jeon and Kim [17]).

Batch reactors are conventionally used for producing bioactive compounds from sea-food processing wastes using enzyme hydrolysis. However, batch reactors have some drawbacks, such as: low productivity because the enzyme is used only once, variability in product characteristics and quality due to batch-to-batch differences, the long times needed which also leads to low productivity, large space requirements and inability to obtain the final product instantly and continuously [16]. The development of the membrane bioreactor overcame many of these problems. A membrane bioreactor integrates a reaction vessel with a membrane separation system as shown in Figure 1. It is composed of two main systems: a bioreactor unit that is responsible for biodegradation of raw materials and the membrane unit that is responsible for the separation of desired bioactive molecules according to molecular size. Various enzymes can be used depending on the desired final products. Compounds with specific molecular weight distributions can be obtained by using membranes with the appropriate molecular weight cutoff (MWCO) or pore size distribution.

The membrane bioreactor also allows the simultaneous recycle of the enzyme and the separation, fractionation and/or concentration of bioactive product molecules. The substrate (sea-food waste) is fed continuously into the reaction vessel and product is continuously withdrawn through the membrane. The advantages of such a system include improved utilization of the enzyme since it is continuously recycled, the product stream (the permeate) is free of suspended matter, product properties (such as molecular weight distribution and clarity) are more consistent and uniform resulting in products of high quality, and the process is continuous, leading to higher productivity.

Membrane bioreactors may appear to be more expensive due to the cost of the membrane unit. However this can usually be justified by its higher productivity, better utilization of the enzyme, superior product quality and no additional downstream clarification is needed. To minimize costs, the membrane unit should be optimized in terms of operating parameters such as pressures, temperature, velocity and concentration. The membrane configuration should be properly selected (e.g., hollow fibers *vs.* spiral wound modules) as well as the separation properties of the membrane. Fouling and concentration polarization should also be minimized by using the appropriate membrane chemistry and operating conditions. Fouling is a result of blocking of membrane pores by feed and/or product compounds. If high-molecular weight compounds such as the substrate are a cause of fouling, this can be alleviated by introducing a pre-hydrolysis step before passing the substrate through the main bioreactor unit. Pre-hydrolysis may also reduce the viscosity of the mixture and enhance or stabilize the enzymatic reaction in membrane bioreactor unit.

### 2.1. Selection of Optimal System Configuration

A membrane bioreactor system consists of two separate parts: a reaction vessel to conduct the reaction and a membrane unit for separation of the peptides and recycle of the enzyme and unconverted substrate. Several parameters have to be considered in each part to run the system effectively. Suitable enzymes, optimum concentrations of substrate and enzyme, as well as operating conditions such as pH, temperature, *etc.*, must be identified before starting the system. The raw materials should also be appropriately pretreated or prepared to minimize operational problems [7,8,18,19].

#### 2.1.1. Membranes

Various membranes have been used depending on availability, suitability and the desired final products [20,21]. Kuroiwa *et al.* [19] used a model UHP-62K system with polysulfone flat-sheet UF membranes of 2000 MWCO from Advantec Co., Japan for the production of COS. Jeon and Kim [17] used a series of membranes with different MWCO of 10 kDa, 5 kDa and 1 kDa for the production of COS. A similar series of membranes with different molecular cut-off (10 kDa, 5 kDa and 1 kDa) were used to produce angiotensin I converting enzyme (ACE) inhibitory peptides from Alaska pollack using a three-step recycling membrane reactor [8]. The separation of fatty acid/triacylglycerol was done with a hydrophilic regenerated cellulose membrane [22].

#### 2.1.2. Enzymes

Chitosanase derived from *Bacillus pumilus* has been used in many studies for the production of COS by membrane bioreactors. Optimum temperature and pH were 35 °C and 5.6 respectively. Alcalase, Neutrase, trypsin, chymotrypsin, Pronase and Collagenase have been used for preparation of peptides from sea-food processing wastes [8]. Optimum temperature and pH for Alcalase, Pronase and Neutrase was 50 °C and 8.0 while Collagenase showed optimum reactivity at 37 °C and pH 7.5. Neutrase is used to partially hydrolyze the substrate and a specific sn-1 sn-3 hydrolytic lipase is used for lipid hydrolysis. Immobilized 1,3-specific lipase IM60 (Lipozym IM) was used for re-esterification. Optimum temperature was 35 °C for Neutrase and a membrane with a MWCO of 10 kDa was used.

## 3. Chitooligosaccharides

**Figure 2:**
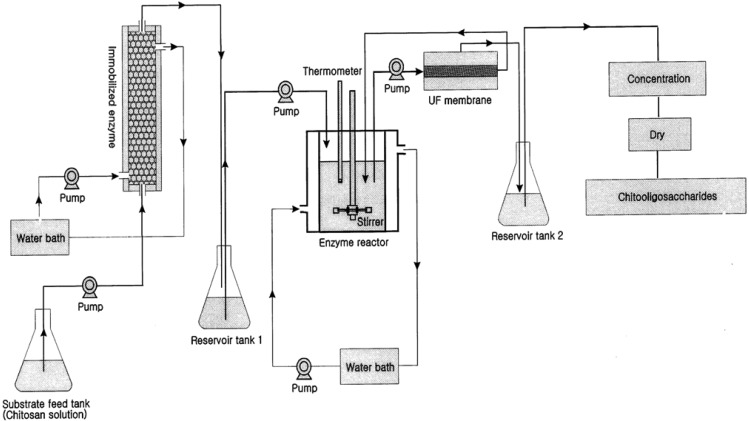
Schematic diagram of the dual reactor system used for continuous production of chitooligosaccharides (Adapted from Jeon and Kim [31]).

The production of COS from chitosan is one of the premium applications of membrane bioreactors in sea-food applications. Crustacean shells and shellfish wastes from sea-food processing plants are an attractive source of bioactive compounds which could potentially be used in the food and pharmaceutical industries [23,24,25,26,27]. Shell wastes are currently utilized for commercial-scale production of chitin as well as for the production of chitosan and COS. There are two methods for production of COS: enzymatic hydrolysis using chitosanase and chemical hydrolysis using acids [28,29]. Chemical hydrolysis is relatively harsh and non-selective and thus the product contains relatively large amounts of impurities including D-glucosamine and chitosan monomers [30]. However, enzymatic hydrolysis in a membrane bioreactor has several advantages with regard to product specificity. Kuroiwa *et al.* [18] obtained the desired target biomolecules (pentamers and hexamers of COS) by proper control of the hydrolysis reaction and using optimal enzyme concentrations. Further, the size of the final products can be controlled by the pore size of the membranes use in the membrane bioreactor system. Their final product concentration of pentamaers and hexamers was 2.6 g/L. The membrane bioreactor was more effective over other systems for the production of COS by hydrolysis of chitosan. Jeon and Kim [31] introduced a new system using dual reactors for producing COS by combining a column reactor packed with immobilized enzymes with an ultrafiltration membrane bioreactor (Figure 2).

### 3.1. Antimicrobial Activity

**Figure 3:**
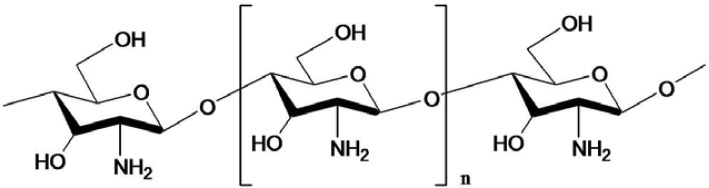
Structure of chitooligosaccharide.

Chitin, chitosan and COS possess a variety of bioactivities (Table 1) and desirable properties like non-toxicity, biocompatibility and biodegradability [32,33,34,35]. Chitosan contains less acetyl groups than chitin and can be prepared either by chemical or microbiological treatments [36]. The structure of COS is given in Figure 3. It is believed that the antibacterial action is due to adsorption of the positively charged quaternary ammonium groups onto the negative charge of bacterial cell surfaces and membrane with subsequent disruption of membrane integrity [37]. Uchida *et al.* [38] found that the inhibition of the activity of fungus and bacteria by COS with higher degrees of polymerization (DP) was much stronger than those by chitosan and COS with lower DP. Further, their inhibitory effects increased with increasing degree of deacetylation. Jeon and Kim [39] confirmed those results with three kinds of COS that were produced and isolated using a membrane bioreactor. COS with molecular weights of 5,000–10,000 Da showed strong antimicrobial activity on the tested pathogens among them [39]. Later, they produced COS with a DP of 3–6 by the same methods, which showed a higher inhibitory effect on *E. coli* with increasing concentration: a 0.5% COS solution completely inhibited the growth of *E. coli*. The relationship between antimicrobial activity and molecular weight of COS was evaluated with different inoculums levels and various concentrations with *E. coli* and *Staphylococcus aureus* in foods [40].

**Table 1:** Biological activities of chitooligosaccharides produced by membrane bioreactors.

**Bioactivities**	**Reference**
Antioxidant	Fernandes *et al.* [41]; Je *et al.* [42]
Antimicrobial	Sajonsang *et al.* [33]; Zhong *et al.* [34];
	Arten *et al.* [35]; Jeon *et al.* [39]
Anticancer	Karagozlu *et al.* [43]; Harish & Thanranatha [44]
Enzyme inhibition	Park *et al.* [45]; Ngo *et al.* [46]
Calcium bioavailability	Jung *et al.* [47]
Anticoagulant	Park *et al.* [48]; Huang *et al.* [49]

### 3.2. Anticancer and Antitumor Activity

COS have been reported to inhibit the growth of tumor cells through its immune-enhancing effects. This occurs not via the direct killing of tumor cells, but by increased production of lymphokines leading to manifestation of antitumor activity through proliferation of cytolytic T-lymphocytes [50]. Aminoderivatized COS was able to inhibit the proliferation of the dose of human gastric adenocarcinoma cells in a time-dependent manner [43]. Three kinds of water-soluble aminoderivatized COS (aminoethyl-COS, dimethyl aminoethyl-COS and diethyl aminoethyl-COS) were prepared and all derivatives have shown promising potential as cancer chemopreventive agents. The antitumor mechanism of COS was probably related with induction of lymphocyte factor, thus increasing T-cell proliferation to produce the tumor inhibitory effects. Suzuki *et al.* [51] analyzed the changes of splenic cells in cancerous mice and concluded that COS enhance acquired immunity by accelerating T-cell differentiation to increase cytotoxicity and maintain T-cell activity. In another study, Harish and Thanranathan [44] reported that COS containing a mixture of pentamers and hexamers may engage in angion inhibition and antitumor activities. Anticancer activity of differently charged COS was evaluated using three cell lines including HeLA, Hep3B and SW480 [52]. Further, irrespective of whether they were positive or negative charges, highly charged COS derivatives could significantly reduce cancer cell viability, perhaps due to necrosis as revealed by DNA-fragmentation and fluorescence microscopic observations.

### 3.3. Inhibition of Enzymes

Chitosan and their derivatives including COS also act as ACE-inhibitors. ACE (angiotensin-converting enzyme) is associated with hypertension. Chloride ions can activate the angiotensin converting enzyme but chitosan or COS can bind to chloride and reduce this complication. The ACE inhibitory activity of chitosan and its derivatives depends on the degree of deacetylation [45]. COS with a low degree of deacetylation exhibited higher ACE inhibitory activity. The activity of COS can be enhanced by changing the structure of COS via addition of various groups. For example, Huang *et al.* [53] added the carboxyl group [–COCH_2_CH_2_COO^−^] to COS and obtained a structure similar to Captopril, which is a commercial antihypertensive agent. This new COS-derivative enhanced ACE inhibitory activity markedly. The activity was competitive via obligatory binding sites of the enzymes. Ngo *et al.* [46] substituted a hydrogen atom at the C-6 position of pyranose with an aminoethyl group and obtained a COS derivative of with strong ACE-inhibitory activity [46].

COS also mediate the inhibition of some important enzymes such as β-secratase, prolyl endopeptidase (PEP) and acetylcholinesterase (AChE) which are involved in neurodegenerative diseases such as Alzheimer's and Parkinson's. The β-secratase inhibitory potential of COS were directly correlated with their degree of deacetylation (DD) and molecular weight [54], with low and medium molecular weight COS having significant inhibitory activity. Enzymes such as PEP and AChE could also be inhibited by COS [55,56]. These observations indicate that COS could be used to treat these type of diseases.

### 3.4. Fat and Cholesterol Lowering Effects

The cholesterol lowering ability of COS was investigated by measuring the levels of serum lipids, antioxidant enzyme activities and lipid peroxidation in rats fed with high cholesterol diets for four weeks [57]. COS supplemented rats had significantly lower serum total cholesterol, LDL cholesterol and triglyceride levels while increasing the relative HDL cholesterol level. COS were able to decrease the cholesterol level in the liver and it could prevent the formation of fatty liver by the action of hepatotrope poison. Several hypotheses have been suggested to explain cholesterol lowering activity of COS. One suggests that it is due to ionic binding of COS with bile salt and bile acid that inhibits micelle formation during the digestion process [58]. Another hypothesis is that chitosan and its oligomers can directly trap lipids and fatty acids [59]. Animal studies indicate that COS increase the excretion of neutral sterol and undigested dietary fats, thus lowering cholesterol levels in the body. Zhou *et al.* [60] have shown that different chitosan preparations were able to bind with bile acids and triglycerides and thus perhaps reduce the risk of diseases due to an increase of fat in the body. Liao *et al.* [61] showed that both water soluble and insoluble chitosan supplementation over a period of 8 weeks lowered the blood lipid in elderly hyperlipidemic patients.

### 3.5. Anticoagulant Activity

Heparin is a well-known antithrombiotic agent that has been used clinically for a long period of time. However, it has some disadvantages such as inefficiency in antithrombin deficient patients, poor bioavailability and variable dose response. Sulfated chitosan oligosaccharide derivatives have been studied as a possible alternative [48,49]. A higher degree of sulfation has a beneficial effect on anticoagulant activity with respect to thrombin time. The arrangement of sulfate groups in the main skeletal also had a tremendous influence on activity. A sulfate group at the C-6 position prolongs thrombin time and activated partial thromboplastin time [48]. Modification of COS structure by adding *N*-propanoyl, *N*-hexanoyl and *N*,*O*-quaternary groups may increase thromboplastin time [49]. Ionic attraction between negatively charged red blood cell membranes and positively charged groups in COS may have caused higher activity.

## 4. Bioactive Peptides

**Figure 4:**
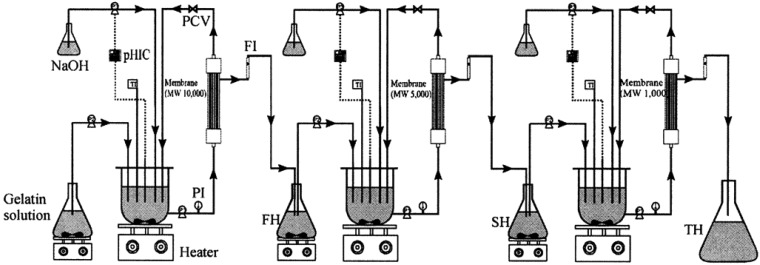
Three-step recycling membrane bioreactor for the production and separation of enzymic hydrolyzates of Alaska Pollack skin gelatin. TI, temperature indicator; PI, pressure indicator; FI, flow indicator; P1, recycling pump; P2, feed pump; P3, NaOH pump; PCV, pressure control valve; pHIC, pH indicator controller; FH, first hydrolysates; SH, second hydrolysates; TH, third hydrolyzate (Adapted from Byun and Kim [8]).

Discarded fish bones and cutoffs may contain considerable amounts of muscle proteins which are nutritionally valuable and easily digestible with a well-balanced amino acid composition [62]. Enzyme hydrolyzates of proteins from seafood wastes exhibit several physicochemical properties and biological activities [63]. The molecular weight of peptides has a large effect on their biological properties and activities and it is desirable to develop methods to produce and separate peptides with different molecular weights. Membrane bioreactors using ultrafiltration membranes of the appropriate molecular weight cutoffs have been effectively used to produce such peptides from seafood wastes [7]. Enzymes used typically are chymotrypsin, trypsin, Alcalase and Neutrase. Byun and Kim [8] used a three-step recycling membrane reactor (Figure 4) to produce peptides having several discrete molecular weights using consecutive proteolytic digestions using three different enzymes followed by fractionation of the hydrolyzate using various membranes. Many studies have confirmed the suitability of this method to obtain low molecular weight peptides that show potent bioactivities [64,65].

Peptides from seafood processing wastes have shown various biological activities including antihypertensive, antioxidative and antiradical activities (Table 2). The molecular weight of the peptide is the single most important factor for determining its biological activity.

**Table 2:** Biological activities of bioactive peptides derived from membrane bioreactor.

**Protein source**	**Bioactivity**	**Reference**
Alaska Pollack skin gelatin	Antioxidant	Kim *et al.* [66]
Hoki frame protein	Antioxidant	Je *et al.* [67]
Alaska Pollack frame protein	Antioxidant	Je *et al.* [68]
Conger eel muscle protein	Antioxidant	Ranatunga *et al.* [69]
Hoki frame protein	Antioxidant	Kim *et al.* [70]
Jumbo squid skin gelatin	Antioxidant	Mendis *et al.* [71]
Fish protein hydrolyzate	Anticoagulant	Rajapakshe *et al.* [9]
Alaska Pollack frame protein	Antihypertensive	Je *et al.* [68]
Yellow fin sole frame protein	Antihypertensive	Jung *et al.* [72]
Alaska Pollack back bone	Calcium bioavailability	Jung *et al.* [73]

### 4.1. Antioxidant and Antiradical Activities

Peptides derived from fish protein have significant antioxidant activities in a variety of oxidative systems [7,74,75]. This activity can be the result of specific scavenging or inhibition of radicals formed during lipid peroxidation, or scavenging of oxygen-containing compounds, or chelating of metal ions [74,76]. Production of fish protein hydrolysates with antioxidant properties will enable production of protein-enriched and oxidatively stable seafood. Cod frame protein hydrolyzate that had been separated into several fractions based on molecular size showed excellent antioxidant activity based on the thiobarbituric acid assay [7]. A protein hydrolyzate obtained from defatted round scads mince containing high amounts of arginine and lysine also exhibited significant antioxidant activity against 2,2-diphenyl-1-picrylhydrazyl (DPPH), and good radical scavenging, metal chelating and reducing properties [77]. Bourseau *et al.* [78] used a two-step process using ultrafiltration and nanofiltration to produce four different fractions with different hydrolysis degrees starting with crude fish protein hydrolysates at a fairly high concentration (100 g of dry matter/L).

Chabeaud *et al.* [79] optimized the antioxidative activity of Alcalase-derived saithe hydrolyzate using response surface methodology. Subsequently, they used a 4000 Da MWCO membrane to separate peptides with a MW less than 1 kDa from saithe protein hydrolyzate [80]. Mendis *et al.* [71] reported lipid peroxidation inhibitory and radical scavenging activities of the peptidic fraction obtained from jumbo squid skin gelatin that had a MW less than 3 kDa. Two chains-breaking low molecular weight antioxidant peptides have been obtained from giant squid muscle protein hydrolysates with strong activities against linoleic acid peroxidation system and radial scavenging systems [81]. In accordance with the results revealed by above two studies, Aleman *et al.* [82] also separated peptides from squid gelatin obtained from inner and outer tunics with potent antioxidant activity. The presence of glycosylated peptides may have contributed to the high antioxidant activity of the squid gelatin hydrolyzate in this study.

### 4.2. ACE Inhibitory Activity

Many studies have been confirmed that bioactive peptides separated from various seafood processing wastes using membrane bioreactors were potent ACE-inhibitors. Aleman *et al.* [82] and Zeng *et al.* [83] suggested the average molecular weight for potent ACE inhibitory activity is about 1400. ACE inhibitory activity is also highly depended on the peptide sequence. ACE appears to prefer substrates or competitive inhibitors that mainly have hydrophobic (aromatic or branched side chains) amino acid residues at the three C-terminal positions [84]. One peptide isolated by Aleman *et al.* [82] had Leu and Gly amino acids in the second and third positions from carboxyl-terminus and this seemed to play an important role in ACE-inhibitory activity. The three-step recycling membrane bioreactor system mentioned earlier was used to produce and separate peptides from gelatin extracts of Alaska Pollack (*Theragra chalcogramma*) [8]. These peptides also had potent ACE inhibitory activity. The molecular weight of the peptides (∼1400) was in agreement with that observed by Zeng *et al.* [83]. Je *et al.* [65] confirmed many of the studies that show potent ACE inhibitory activities even for peptides with MW lower than 1 kDa. Yellow fin sole frame protein was hydrolyzed by α-chymotrypsin and fractionated into three ranges of molecular weights with a membrane bioreactor [72]. Peptides with lower molecular weight showed potent ACE inhibitory activity compared to peptides with higher molecular weights. Further, these peptides showed *in vivo* activities by lowering the blood pressure significantly in spontaneously hypertensive rats following oral administration. This is an advantage of fish protein hydrolysates, since some antihypertensive drugs are reported to have side effects including abnormal elevation of blood pressure after administration.

### 4.3. Calcium Bioavailability and Solubilization

Calcium deficiency causes various disorders and diseases in the human body such as osteoporosis, cardiovascular diseases, diabetes and cancer. Bioactive peptides may help to regulate calcium levels and calcium homoeostasis and increase the bioavailability and solubilization of calcium. Jung *et al.* [11] prepared oligophosphopeptides from the bones discarded from industrial processing of *hoki* using heterogeneous enzymes extracted from intestines of carnivorous fish. Those peptides increased calcium retention while increasing the soluble calcium by 41.1 mg/L without formation of insoluble calcium phosphate. In a follow-up study, Jung *et al.* [73] isolated fish bone peptides with a high affinity for calcium from Alaska Pollock using ultrafiltration and were shown to inhibit the formation of insoluble calcium at neutral pH. *In vivo* effects of these peptides were evaluated with overiectomised rats and it was shown that calcium retention was increased and loss of bone mineral was decreased. Low molecular weight peptides with high affinity for calcium were also recovered from hydrolysates of Alaska Pollack backbone wastes. These peptides were as effective as casein phosphopeptide in solubilizing calcium [85].

## 5. Bioactive Lipids

Fish oils are readily available source of n-3 PUFA such as eicosapentaenoic acid (EPA) and docosahexaenoic acid (DHA) which plays an important role in the prevention of a number of diseases, including atherosclerosis, coronary heart disease, hypertension, inflammation, hypotriglyceridemic effect, allergies and diabetes [86,87,88]. The use of membranes in lipid processing has attracted great interest in recent years since lipids that are heat labile can be preserved and concentrated with minimal changes to the lipid [18]. Most studies in this area have been on hydrolysis of oils and fats [89], the synthesis of acylglycerols with two phase membrane reactors [90] and epoxidation of fatty acids [91]. Sahashi *et al.* [92] combined solvent extraction with membranes to fish oil triglycerides from free fatty acids. An ethanol solvent (75% aqueous ethanol) was added to the fish oil which contained triglycerides with polyunsaturated fatty acids (PUFAs) and free fatty acids (FFA). Several flat-sheet membranes were tested for their ability to separate the oil from the solvent phase which contained the FFA. A hydrophilic polyimide ultrafiltration membrane with 20 kDa molecular cut-off was found to be the most suitable for the separation of the solvent phase as permeate. A rotating disk membrane was effective in a large-scale operation for getting PUFA-rich glycerides without oil quality deterioration. Xu *et al.* [93] used membrane bioreactor to separate medium chain triacylglycerols and n-3 PUFAs from fish oil. In their study lypozyme was used as the biocatalyst to accelerate the reaction in the bioreactor. The medium chain fatty acids were separated by 10 kDa and 2 kDa MWCO membranes.

Linder *et al.* [22] conducted the enzymatic hydrolysis using stereospecific sn-1 sn-3 hydrolytic lipase from *Aspergillus oryzae* followed by membrane filtration. The substrate was then partially hydrolyzed by the protease Neutrase to obtain most of the oil after centrifugation of the mixture. Immobilized 1,3-specific lipase IM60 (Lysozyme IM) was used for re-esterification. The incubation of the mixture was carried out at 37 °C, pH 7 for 24 h under stirring at 800 rpm. Lipase hydrolysis was carried out in a batch reactor blanketed with nitrogen. Membrane separation increased the PUFAs from 30.4% in crude oil to 43.3% in permeate. D'Arrigo *et al.* [90] have used a membrane bioreactor for hydrolysis as well as transphosphotidylation reactions. In many studies, lyposyme has been used as the catalyst to separate eicosapentaenoic acid (EPA) and docosahexaenoic acid (DHA) in fish oil [94].

Fish oil intervention resulted in a significant increase in platelet phospholipids EPA content, decrease in arachidonic and γ-linolenic acid levels and significant decrease in fasting and postprandial triacylglycerol levels [87]. Long chain PUFAs are considered as essential for the growth and development of infants and they have been included in food supplements in the form of concentrated fish oil. Hence, there is a marked demand for marine fatty acids as a health food and for use as a food supplement.

## 6. Enzyme Separation by Membrane Bioreactors

Proteases are widely used in the food industry for the production of protein hydrolysates, meat tenderization and fermentation [95]. Proteases such as trypsin and chymotrypsin from yellowfin tuna spleen extract (one of the best sources, according to Jantaro [96]) were separated by ultrafiltration using regenerated cellulose membranes with 30 kDa and 100 kDa MWCO membranes [97]. The limiting fluxes at cross-flow rate 120, 240 and 360 L/h for the 30 kDa membrane were 17.3, 43.9 and 54.7 L/m^2^ h, respectively and the limiting fluxes at the same flow rate for 100 kDa membrane were 34.1, 51.1 and 68.4 L/m^2^ h, respectively. In recent years, ultrafiltration has extensively been used for the separation of a wide range of compounds, including proteases from surimi wash water [98], proteases and protein hydrolysates from fish viscera [99].

## 7. Summary and Conclusions

Seafood processing wastes have great potential for the manufacture of bioactive substances. Even a few value-added compounds recovered from such wastes are economically more important than the target products themselves. However, their suitability as bioactive compounds and their pharmacological and nutraceutical values have not yet been properly evaluated and need to be studied more before considering them as nutraceuticals. Development of novel methods to produce and recover them is necessary. Membrane bioreactor technology can be used to separate and fractionate these bioactive compounds from marine wastes and will lead to the development of more profitable processes, thus giving rise to many great opportunities in the marine industry.

The increased focus of membrane manufacturers on membrane bioreactor applications is likely to continue in the future and result in more cost-effective systems. Better membranes and more efficient modules are also likely additional future developments. Introduction of low-cost, low-energy bioreactor technology is necessary to increase its utilization. A better understanding of membrane fouling is needed and new strategies need to be developed to concentrate the bioactive materials (involving novel reactor designs), and to increase the immobilization of enzymes in the reactor (use of carrier materials).
